# Mechanical chest compressions in the coronary catheterization laboratory to facilitate coronary intervention and survival in patients requiring prolonged resuscitation efforts

**DOI:** 10.1186/s13049-016-0198-3

**Published:** 2016-01-21

**Authors:** Henrik Wagner, Bjarne Madsen Hardig, Malin Rundgren, David Zughaft, Jan Harnek, Matthias Götberg, Göran K. Olivecrona

**Affiliations:** Department of Cardiology, Lund University, Lund, Sweden; Physio-Control Sweden/Jolife AB, Lund, Sweden; Department of Anaesthesiology and Intensive Care, Lund University, Lund, Sweden

**Keywords:** Cardiac arrest, Resuscitation, Mechanical chest compressions, Percutaneous coronary intervention, Survival

## Abstract

**Background:**

Resuscitation after cardiac arrest (CA) in the catheterization laboratory (cath-lab) using mechanical chest compressions (CC) during simultaneous percutaneous coronary intervention (PCI) is a strong recommendation in the 2015 European Resuscitation Council (ERC) guidelines. This study aimed at re-evaluating survival to hospital discharge and assess long term outcome in this patient population.

**Methods:**

Patients presenting at the cath lab with spontaneous circulation, suffering CA and requiring prolonged mechanical CC during cath lab procedures between 2009 and 2013 were included. Circumstances leading to CA, resuscitation parameters and outcomes were evaluated within this cohort. For comparison, patients needing prolonged manual CC in the cath lab in the pre-mechanical CC era were evaluated. Six-month and one year survival with a mechanical CC treatment strategy from 2004 to 2013 was also evaluated.

**Results:**

Thirty-two patients were included between 2009 and 2013 (24 ST-elevation myocardial infarction (STEMI), 4 non-STEMI, 2 planned PCI, 1 angiogram and 1 intra-aortic counter pulsation balloon pump insertion). Twenty were in cardiogenic shock prior to inclusion. Twenty-five were successfully treated with PCI. Median mechanical CC duration for the total cohort (*n* = 32) was 34 min (range 5–90), for the 15 patients with circulation discharged from the cath-lab, 15 min (range 5–90), and for the eight discharged alive from hospital, 10 min (range 5–52). Twenty-five percent survived with good neurological outcome at hospital discharge. Ten patients treated with manual CC were included with one survivor.

**Discussion:**

Eighty-seven percent of the patients included in the mechanical CC cohort had their coronary or cardiac intervention performed during mechanical CC with an 80 % success rate. This shows that the use of mechanical CC during an intervention does not seem to impair the interventional result substantially. The survival rate after one year was 87 %.

**Conclusions:**

Among patients suffering CA treated with mechanical CC in the cath-lab, 25 % had a good neurological outcome at hospital discharge compared to 10 % treated with manual CC. Long term survival in patients discharged from hospital is good.

## Background

Since 2004, we have routinely used a mechanical chest compression (CC) device in cardiac arrest (CA) situations in the coronary catheterization laboratory (cath-lab) when initial advanced resuscitation efforts have failed to obtain return of spontaneous circulation. A mechanical CC device can successfully be used to overcome the difficulties of performing manual CC during simultaneous percutaneous coronary intervention (PCI) [1]. There have been an increasing number of publications describing favourable outcomes with this treatment option, mostly small cohort studies and case reports [2–7]. We have previously documented a 25 % survival rate in cerebral performance category 1 or 2 at hospital discharge using this treatment strategy [8]. Since 2015, the use of mechanical CC in CA situations in the cath-lab during simultaneous PCI is strongly recommended in the European Resuscitation Council (ERC) guidelines [9].

When implementing a new treatment strategy, it is important to evaluate the results over a long period of time. One of the difficulties with new treatment options is that they are often introduced without critical evaluation such as a randomized trial and without an organized implementation [10]. Over the years of using mechanical CC in the cath-lab in resuscitation efforts in combination with simultaneous PCI, we have noted several practical short-comings in the work-flow. This has led to the development of a more structured and more tightly conducted approach, which has been described in detail elsewhere [11]. We therefore continued to evaluate both short and long term outcomes in patients suffering CA treated with mechanical CC during an invasive cardiac/coronary procedure in a prospective manner between 9 April 2009 and 9 April 2013.

Other important factors are patients survival to hospital discharge and long term survival after CA in the cath-lab when they have been treated with prolonged advanced resuscitation efforts during an invasive cardiac/coronary procedure. Ehlenbach et al. analysed the outcomes of CA, in individuals > 65 years of age suffering in-hospital CA, where the survival to discharge was 18.3 % [12]. Girotra et al. studied in-hospital CA with an overall survival rate of 17 % to discharge from hospital, with survival rates increasing from 13.7 to 22.3 % at the end of the study [13]. However, CA cases occurring during a procedure in the operating room, in procedural suites or in the emergency department, were excluded [13]. A recently published study on in-hospital CA found a survival rate to discharge from hospital of 18.4 % [14]. Further, in a Swedish study, the survival rate to discharge in in-hospital CA was found to be 37 % and 1-year survival among discharged patients was 84 % [15]. Another study focusing on in-hospital CA, in an elderly cohort, > 65 years of age, concluded that among the patients discharged from hospital, 59 % were alive 1 year after discharge [16]. In our previous patients series were mechanical CC during PCI was used [8] we did not explore the long term survival outcome. Therefore these patients were included in the long term follow up in the this publication.

The aim of this prospective study was to analyse circumstances leading to CA and resuscitation parameters, to re-evaluate survival to hospital discharge and to assess 6-month and 1 year survival in this patient population who suffer CA and require prolonged resuscitation with mechanical CC in combination with an invasive cardiac/coronary procedure.

## Methods

This prospective study was performed between 9 April 2009 and 9 April 2013 at the cath-lab at Skane University Hospital, Lund, Sweden. This is a tertiary centre in southern Sweden that performs PCIs 24 h a day, 7 days a week, and serves a population of 1.2 million. This study was approved by the local ethics review board (667/2009) and informed consent was obtained from survivors or from family members.

Among those who suffered a CA in the cath-lab, patients were included if resuscitation efforts has not solved the CA in a few minutes by defibrillation or drug treatment and there was consensus among the attending cardiologist, anaesthesiologist and the interventionist that mechanical CC was indicated. Cardiac arrest treatment prior to inclusion was performed according to the structural approach described previously, which also describes practical handling in the cath-lab setting including how to manoeuver the image amplifier, when and how to deploy the Mechanical CC device on the patient [11]. The reason for referral to the cath-lab for those who were included in the study was either a diagnostic coronary angiogram in a coronary stable state, non-ST-elevation myocardial infarction (non-STEMI), elective planned PCI, insertion of an intra-aortic balloon counter pulsation device and for primary PCI in patients suffering a STEMI.

For mechanical CC, the LUCAS™2 chest compression system (Physio-Control/Jolife AB, Lund, Sweden) was used. The patient cohort was evaluated in four outcome groups: patient characteristics for the whole group, for patients who expired in the cath-lab, for patients discharged from the cath-lab and for patients discharged from hospital. The predefined endpoints were return of spontaneous circulation when leaving the cath-lab, and hospital discharge in cerebral performance category 1 or 2 [17]. The cause of the referral to the cath-lab, culprit lesion, circulatory state at the time of arrival to the cath-lab and rhythm at the time of the occurrence of the CA, were assessed in the 4 outcome groups. The number of PCIs during mechanical CC was assessed and successful PCI was defined according to the thrombolysis in myocardial infarction (TIMI) flow [18]. Treatment times with mechanical CC were calculated for all groups and compared across the four outcome groups. The use of vasoactive drugs was assessed.

As a comparison, outcome for 10 consecutive patients suffering CA who needed prolonged resuscitation with manual CC in the cath lab (from 1999 to 2003, the time period prior to the start of using mechanical CC in our lab) were analysed using the local hospital CA registry and medical files. Inclusion criteria were the same as for those treated with mechanical CC (i.e. patients suffering CA where initial early resuscitation efforts failed).

Long term follow up was done for patients discharged from hospital both from the previous retrospective registry study (1 January 2004 to 31 December 2008) [8] and those discharged from hospital in the current study.

### Statistical methods

Continuous data are presented as mean ± SD and median and range as appropriate. Categorical variables are presented as numbers or percentages. For non-parametric statistics, the Mann-Whitney *U*-test was used for calculating differences between the outcome groups, in age and mechanical CC time. A *p*-value < 0.05 was considered significant.

## Results

Thirty-two patients were included during this study period. For patient demographics see Table 1. Patient characteristics such as the indications for cath-lab procedure, culprit lesion, circulatory state upon arrival at the cath-lab, and rhythm when the CA occurred, are presented in Table 2.

**Table 1:** Patient demographics, concomitant diseases, smoking habits and previous coronary interventions

	All patients	Expired Cath-lab	Discharged Cath-lab	Discharged Hospital
Patient History	*n* = 32 (%)	17 (53)	15 (47)	8 (25)
Age	71 ± 13	73 ± 10	68 ± 15	68 ± 19
Gender (male)	20 (63)	11	9	4
Hypertension	18 (56 %)	9	9	7
Diabetes	8 (25 %)	6	2	2
Hyperlipidaemia	9 (28 %)	7	2	2
Smoking/X-smoker	14 (44 %)	7	7	4
Previous MI	9 (28 %)	4	5	4
Previous PCI	3 (9 %)	1	2	2
Previous CABG	4 (13 %)	3	1	1

*Cath-lab* coronary catheterization laboratory, *MI* myocardial infarction, *PCI* percutaneous coronary intervention, *CABG* coronary artery by-pass grafting

**Table 2:** Indication for referral to the coronary catheterization laboratory, culprit lesion, circulatory state at arrival in the coronary catheterization laboratory, rhythm at the time of the cardiac arrest

	All patients	Expired Cath-lab	Discharged Cath-lab	Discharged Hospital
	n = 32 (%)	17 (53)	15 (47)	8 (25)
Indication for cath-lab procedure				
STEMI	24 (75)	15	9	4
non-STEMI	4 (13)	1	3	2
Elective PCI	2 (6)	1	1	1
Other	1 (3)	0	1	1
Angiogram	1 (3)	0	1	0
Culprit lesion in coronary patients				
LM	10 (31)	6	4	2
LAD	12 (38)	7	5	2
LCx	2 (6)	0	2	2
RCA	6 (19)	4	2	1
Other	2 (6)	0	2	1
Circulatory state at the arrival to the cath-lab				
Cardiogenic shock	20 (62)	12	8	2
Initial rhythm at cardiac arrest				
VT/VF	5 (16)	1	4	2
PEA	22 (69)	14	8	4
Asystole	5 (16)	2	3	2

*Cath-lab* coronary catheterization laboratory, *STEMI* ST-elevation myocardial infarction, *non-STEMI* non-ST-elevation myocardial infarction, *PCI* percutaneous coronary intervention, *LM* left main coronary artery, *LAD* left anterior descendent coronary artery, *LCx* left circumflex coronary artery, *RCA* right coronary artery, *VT* ventricular tachycardia, *VF* ventricular fibrillation, *PEA* pulseless electrical activity

In one specific patient, the reason for referral for the intra-aortic balloon counter pulsation insertion was therapy resistant ventricular tachycardia with cardiogenic shock. In the patients referred for planned PCI and non-STEMI, complications such as, for example, thrombus formation, vessel rupture and dissection caused the CA. One of the patients with non-STEMI was in cardiogenic shock at the time of arrival at the cath-lab. The patient, who was referred for an elective pre-operative coronary angiogram for surgery on the aortic valve, deteriorated into pulseless electrical activity due to aortic stenosis and reduced systolic left ventricular function.

Seventeen patients expired in the cath-lab. Fifteen left the cath-lab with circulation, of whom eight were discharged from hospital with cerebral performance category 1–2. During the study period (9 April 2009 – 9 April 2013), 8738 patients were admitted to the cath-lab for an invasive cardiac/coronary procedure. In total, 3368 patients were evaluated with a coronary angiogram only and 5370 patents were treated with PCI (acute or elective) whereof 2728 were treated for STEMI. Of these, 116 patients were in cardiogenic shock when admitted to the cath-lab. There was no statistical age difference between the patients who expired in the cath-lab and those who were discharged from the cath-lab with circulation (*p* = 0.37) and those discharged from hospital (*p* = 0.64). Successful PCI defined as TIMI-II-III or < 50 % residual stenosis, PCI during mechanical CC and treatment time with mechanical CC are presented in Table 3. There was a statistically significant difference in time with mechanical CC when comparing patients who expired in the cath-lab to those discharged from the cath-lab with circulation (*p* = 0.02) and to those discharged from hospital (*p* = 0.004). At least one vasoactive drug (norepinephrine, epinephrine or dobutamine) was administered either intermittently or as a continuous infusion) to 29 patients, and the majority received a combination of these drugs during the procedure.

**Table 3:** Coronary catheterization laboratory procedural data

	All patients	Expired Cath-lab	Discharged Cath-lab	Discharged Hospital
Procedural data	*n* = 32 (%)	17 (53)	15 (47)	8 (25)
Angiography during MCC	5 (16)	2	3	2
PCI during MCC	27 (87)	16	11	6
PCI successful	25 (81)	12	13	6
PCI unsuccessful	6 (20)	5	1	1
Use of concomitant IABP	12 (38)	3	9	4
CC- time	34 (5–90)	42 (10–75)	15 (5–90)	10 (5–52)
Thoracic surgery	4 (13)	0	4	2

*Cath-lab* coronary catheterization laboratory, *PCI* percutaneous coronary intervention, *MCC* mechanical chest compression, *IABP* intra-aortic counter pulsation pump, *CC* chest compression times are presented as median minutes (range)

### The manual CC treated group

Ten patients (eight men) with a mean age of 68 ± 6 years suffered CA and required prolonged advanced resuscitation efforts with manual CC in the cath-lab between 1 January 1999 and 31 December 2003. Eight of these were referred due to a STEMI; one patient had a non-STEMI, and one patient had developed a ventricular septum defect due to a STEMI a few days earlier. Seven patients were in cardiogenic shock when admitted to the cath-lab. Two patients had a shockable rhythm at the time of the CA. Six patients were treated with PCI during manual CC, with 50 % PCI success rate. The median time with manual CC for the whole group (*n* = 10) was 20 min (range 15–75 min), 20 min (range 15–75) for those who expired in the cath-lab (*n* = 6), 25 min (range 15–60) for those discharged from the cath-lab (*n* = 4), and 15 min for the patient discharged from hospital in cerebral performance category 1 (*n* = 1). Comparison of data relating to patient history, indication for cath-lab procedure, culprit lesion, procedural data and the initial rhythm at CA between the patients treated with mechanical CC and those treated with manual CC’s can be seen in Table 4.

**Table 4:** Patient history, concomitant diseases, smoking habits and previous coronary interventions. Indication for referral to the coronary catheterization laboratory, culprit lesion, circulatory state at arrival to the coronary catheterization laboratory, rhythm at the time of the cardiac arrest. Coronary catheterization laboratory procedural data. Comparing those treated with Mechanical CPR and those treated with manual CPR

	Mechanical CC in the lab	Manual CC in the lab
	*n* = 32	*n* = 10
Patient History		
Age	71 (±13)	68 (±6)
Gender (male)	20	8
Hypertension	18	1
Diabetes	8	2
Hyperlipidemia	9	1
Smoking/X-smoke	14	2
Previous MI	9	2
Previous PCI	3	1
Previous CABG	4	1
Indication for cath lab procedure		
STEMI	23	8
non-STEMI	4	1
Elective PCI	2	0
Tamponade	0	0
Other	2	1
Angiogram	1	0
Culprit lesion		
LM	10	1
LAD	12	5
LCx	2	1
RCA	6	1
Other	2	2
Procedural data		
Angiography only	5	1
PCI successful	25	3
PCI Unsuccessful	6	3
Use of IABP	12	5
CC- time	34 (5–90)	20 (15–75)
Cardiogenic shock	20	7
Initial rhythm at cardiac arrest		
VT/VF	5	2
PEA	22	4
Asystole	5	4

*Cath-lab* coronary catheterization laboratory, *STEMI* ST-elevation myocardial infarction, *non-STEMI* non-ST-elevation myocardial infarction, *PCI* percutaneous coronary intervention, *LM* left main coronary artery, *LAD* left anterior descendent coronary artery, *LCx* left circumflex coronary artery, *RCA* right coronary artery, *VT* ventricular tachycardia, *VF* ventricular fibrillation, *PEA* pulseless electrical activity

### Long term survival

**Fig. 1:**
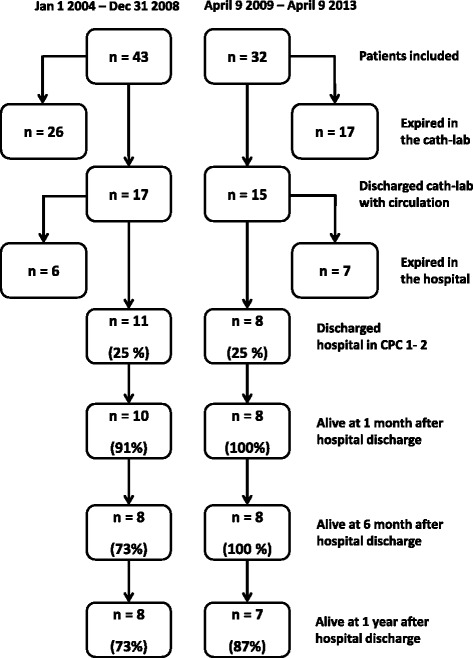
Flow-chart showing the included patients requiring prolonged advanced resuscitation including mechanical chest compressions during percutaneous coronary/cardiac interventions (Cath-lab = coronary catheterization laboratory, CPC = cerebral performance category)

The long term survival for the present patient cohort that were discharged from hospital (8 (25 %)) was, 8 patients that (100 %) survived to 1 month after discharge, 8 (100 %) patients that survived to 6 month and 7 patients (87 %) were alive at 1 year, all in CPC 1–2. In our previous patients series the long term survival in those discharged from hospital (11 patients (25 %)), was 10 patients (91 %) that survived to 1 month after discharge, 8 patients (73 %) that survived to 6 month and 8 patients (73 %) were alive at 1 year, all in CPC 1–2 (Fig. 1).

## Discussion

In this prospective study evaluating the outcomes of patients treated with mechanical CC during a simultaneous cardiac/coronary procedure due to CA where normal advanced resuscitation efforts had failed, we found a 25 % survival rate in cerebral performance category 1 or 2 at hospital discharge. These results verify similar survival results at hospital discharge as our previous retrospective study [8] and lend further support to the current ERC guideline [9]. The structured and more tightly conducted approach used in this second period did show an improvement in team work and resulted in a more calm and success oriented setting [11]. There is an increase in 1 year survival rate from 73 % in the period between 1 January 2004 and 31 December 2008 to 87 % in the current study that might reflect the new approach, but the figures should be handled with precaution since it is small numbers.

In-hospital CA could depend on a broad range of underlying conditions [12–15, 19, 20]. Reported survival rates to discharge from hospital after in-hospital CA vary widely, from 17 to 36 % [12–15, 19, 20] and differences in inclusion and exclusion criteria are important when interpreting the results. In some studies, subgroups such as patients suffering a CA during a medical procedure or in the emergency department [13], or patients < 65 years of age, have been excluded [12]. There are also important differences in background factors such as a high rate of initial shockable rhythm (49 and 39 %) [15, 20]. As a comparison, the study presented here included patients without age restriction suffering CA in the cath-lab, who required prolonged advanced resuscitation efforts including mechanical CC during an intervention, and only 16 % had an initial shockable rhythm. Thus, comparisons with the referred studies are delicate because of the important population differences.

In one registry report covering survival to hospital discharge after a CA in the cath-lab, survival was as high as 65 % [19]. The survival difference compared to our study may be caused by different inclusion criteria. In the report by Herlitz et al. [19], a large proportion of patients suffering a CA may have received one or two defibrillations and/or a few moments of manual CC. The incidence of reperfusion ventricular fibrillation in patients with STEMI treated by primary PCI in the cath-lab in our institution is 1.9 % annually, with a survival rate of 81.7 % at discharge from hospital when defibrillated early [21]. In the current study we have excluded these specific patients: hence making direct comparisons with the study by Herlitz et al. [19] and the study by Demidova et al. [21] in terms of survival are difficult.

In the current study, 27 patients (84 %) had a non-shockable rhythm at the time of the CA. This indicates that coronary ischemic-driven CA in this setting has a large proportion of patients presenting with a rhythm not treatable with defibrillation and that most of the patients with a ventricular fibrillation are easily treated with an early defibrillation as shown by Demidova et al. [21]. A similar percentage of ventricular fibrillation was seen in our previous study [8] and in our historical group treated with manual CC (Table 4). However, these percentages differ from other in-hospital studies, where 51 and 61 % had an initial non-shockable rhythm [15, 20]. Again the cohorts studied in these papers differ, because all patients suffering a CA by any cause are included [15, 20] compared to the highly selected cohort in our current study. However, in the study by Nolan et al. there was a high amount of non-shockable rhythm (72.9 %) and only 16.9 % had an initial shockable rhythm [14]. The cause is unclear, but one explanation might be that 56.6 % of the CAs occurred in a general ward, likely without monitoring [14]. Thus the occurrence of the CA may not be instantly noticed, which may lead to the conversion of an initial ventricular fibrillation or a pulseless ventricular tachycardia to a non-shockable rhythm, but this remains speculative.

Twenty-five out of 31 (80 %) interventions were successful. Considering that 87 % of the interventions were performed during mechanical CC, an 80 % success rate is reasonable compared to the expected 90 % success rate in primary PCI for STEMI [22]. In the previous study, there was a 76 % PCI success rate [8]. Thus, the use of mechanical CC during simultaneous PCI does not appear to reduce PCI results substantially compared to primary PCI for STEMI.

In the 10 patients from the pre-mechanical CC era treated with manual CC, 10 % survived to hospital discharge. One problem comparing historical data is that indications for referral to the cath-lab may have changed over time. This may be reflected by the fact that only 10 patients in four years needed prolonged resuscitation. The DANAMI-2 trial in 2003 showed superiority for primary PCI compared to fibrinolytic therapy in STEMI-patients [23], and Hochman et al. showed a survival benefit in patients in cardiogenic shock treated with early invasive strategy both in the short and long term perspective [24, 25]. The results of these studies may have increased referrals to the cath-lab of patients in a more severe cardio-vascular circulatory condition at greater risk of developing CA.

In the group treated with mechanical CC, only two (25 %) of the patients discharged alive from hospital were in cardiogenic shock compared to 12 (60 %) who expired in the cath-lab. This mortality rate is higher compared to what was reported by Minha et al. where 29 % with cardiogenic shock expired in the cath-lab [26]. In another large registry (patients suffering CA and resuscitated prior to PCI), CA was more common in patients with cardiogenic shock presenting with and without STEMI, where 82 % of the deceased patients in the STEMI-group and 78 % in the non-STEMI group were in cardiogenic shock [27]. One explanation for the high mortality rate in the group with cardiogenic shock might be that when a CA has occurred, the hemodynamic status prior to the CA reflects the severity of the disease, which may be important for achieving the return of spontaneous circulation.

The 1 year survival rate for patients in our previous study and current study discharged from the hospital in cerebral performance category 1–2 was 73 and 87 % respectively. This is higher in comparison to a large retrospective study of elderly patients where the survival rate after in-hospital CA was found to be 58 % at one year [16] but similar to the study from Fredriksson et al. [15]. However, these studies included different causes of CA and survival was analysed at different time points, which makes comparison difficult.

When implementing new technologies in medical settings, it is important that this can be done methodologically and with thorough follow-up [10]. In the case of the implementation of mechanical CC in the cath-lab, there have been no randomized trials. Over the past ten years, we have included 75 patients and show a survival rate of 25 % at discharge from hospital. With this number of patients and reproducible findings of successful interventions, we find the use of mechanical CC devices in the cath lab to be a reliable, safe and a valuable tool in the treatment of CA during PCI.

In an effort to further increase the survival rate for these patients, extracorporeal membranous oxygenation has been used in CA treatment situations with some success [28–30]. If implementing this strategy, the challenge will be to choose the “right” patients. In a Japanese study, the authors found that patients with refractory ventricular fibrillation and pulseless ventricular tachycardia without any evidence of developing signs of multi-organ dysfunction had a favourable outcome when treated with extracorporeal membranous oxygenation [31]. Most of those who died in our current study were in cardiogenic shock at the time of admission to the cath-lab. A reasonable thought is that the patients with cardiogenic shock should be selected for extracorporeal membranous oxygenation or Impella®, perhaps prior to the occurrence of the CA. Both the strategy with Impella® as well with extracorporeal membranous oxygenation for prolonged resuscitation efforts are currently under evaluation in two randomized studies [32, 33].

### Limitations

The study has some limitations. Firstly, it was not a randomized, controlled study. However, performing a randomized study in this setting and comparing mechanical CC to manual CC during simultaneous PCI might be controversial, since it is exceedingly difficult to perform manual CC during simultaneous PCI and the CC provider would be exposed to unacceptably high amounts of X-ray radiation. The numbers of patients treated with manual CC during prolonged CA in the cath-lab are small, which make comparison difficult, but the time period covering the pre-mechanical CC era could not be extended since the in hospital CA registry started in 1999. Having a new treatment strategy for this group of patients in the need of prolonged resuscitation might influence the decision to treat more patients which also can affect results. The control patients were also treated during a time period were poorer post resuscitation care was available, which could have an effect on the long term outcome in this group. Despite the limitation of being a small study (only 0.09 % of the patients who were referred for primary PCI suffered CA and required prolonged resuscitation including mechanical CC), this is the second-largest prospective single centre case series (*n* = 32) describing the use of mechanical CC devices in the cath-lab.

## Conclusion

This study confirms the results of our earlier study, which showed a survival rate at hospital discharge of 25 % in patients treated with mechanical CC during PCI and who arrived at the cath-lab with spontaneous circulation. Furthermore, there was good long term survival rate with good neurological outcome among patients discharged from the hospital.
